# Quality of life in patients with vitiligo: a cross-sectional study based on Vitiligo Quality of Life index (VitiQoL)

**DOI:** 10.1186/s12955-016-0490-y

**Published:** 2016-06-07

**Authors:** Kosar Hedayat, Mojgan Karbakhsh, Maryam Ghiasi, Azadeh Goodarzi, Yousef Fakour, Zahra Akbari, Afsaneh Ghayoumi, Narges Ghandi

**Affiliations:** Autoimmune Bullous Diseases Research Center, Tehran University of Medical Sciences, Tehran, Iran; Department of Community and Preventive Medicine, Tehran University of Medical Sciences, Tehran, Iran; Department of Dermatology, Iran University of Medical Sciences, Tehran, Iran; Department of Psychology, Razi Hospital, Tehran University of Medical Sciences, Tehran, Iran

**Keywords:** Vitiligo, Quality of life, Vitiligo quality of life instrument, VitiQoL, Vitiligo area and score index, VASI, Iran

## Abstract

**Background:**

Vitiligo is a multi-factorial pigmentary skin disorder. Recently, the importance of emotional and psychological issues is proposed in incidence, progression, relapse and remission of vitiligo. There are limited studies conducted in developing countries, which assess life quality of patients with vitiligo. The aim of this study was the application and evaluation of a disease-specific quality of life index in Iranian patients, for the first time.

**Methods:**

This cross-sectional biphasic study was conducted on 25 patients as a pilot and another 173 patients as the main study group, in Razi Hospital, Tehran, Iran, 2013–2014. Persian version of Vitiligo Quality of Life index (VitiQoL) was developed with backward-forward method. Based on the pilot study, the validity and reliability were assessed. The Vitiligo Area and Score Index (VASI), VitiQoL, and their relationship, demographic and clinical characteristic of patients were measured.

**Results:**

The Mean and standard deviation of the VitiQoL score was 30.5 ± 14.5 (range 0–60 in Persian version). There was a significant relationship between VASI score and VitiQoL (*p* = 0.015, *r* = 0.187). Confirmatory factor analysis revealed three important factors within VitiQoL: participation limitation, stigma, and behavior. In subscale analysis based on behavior factor, female patients had poorer quality of life (*p* = 0.02). Concomitant psychiatric problems, e.g. anxiety and depression, were not associated with QOL; however, they were near to being meaningful (*p* = 0.06, *r* = 0.14).

**Conclusion:**

VitiQoL is a valid index in estimating life quality of vitiligo patients and has proper relation to disease severity. Focusing on patient’s life quality is an important entity in the management of vitiligo patients; relevant supportive group-based consultations and therapies are also important arms when approaching vitiligo.

**Electronic supplementary material:**

The online version of this article (doi:10.1186/s12955-016-0490-y) contains supplementary material, which is available to authorized users.

## Background

Vitiligo is an acquired, multi-factorial and usually progressive disorder of melanin production. Vitiligo equally affects males and females as well as all different races. Vitiligo has various onset-age, distribution pattern and progression course. Vitiligo is the most prevalent cutaneous pigmentary disorder. Its prevalence reaches 0.5–2 %, worldwide. The mean age of onset is about 20 years and in 95 % of cases are under the age of 40 [1–4].

The aim of vitiligo treatment is to get better skin appearance, which requires long time, usually 6–18 months. Response to treatment is about 70–75 % and how to choose a treatment modality depends on severity, extent and location of disease [5]. Vitiligo Area Scoring Index (VASI) is one of the most valid indexes for evaluation of disease extent and severity and also it can be measured in all situations [6]. Vitiligo European Task Force (VETF) is the best assessor of treatment efficacy; however, it needs wood lamp and sometimes taking photograph is necessary [7].

Recently, importance of emotional and psychological issues has been raised. Therefore, vitiligo could be considered as a psychosomatic disorder, which means physical and psychological factors concomitantly are involved in appearance, progression, relapse and remission of vitiligo [8]. Vitiligo imposes a large burden on patients’ lives, and many patients suffer from shame and embarrassment, low self-confidence, and social isolation [9–17].

Wrong social beliefs exacerbate the problems of vitiligo patients. As in women, vitiligo may end in divorce and some people believe it is God’s punishment for the sins [18]. In India, sometimes vitiligo is considered as white leprosy, which has a significant stigma [10].

Although vitiligo has a remarkable impact on quality of life (QOL) and brings social stigma, most of the studies on this aspect of vitiligo are based on general cutaneous or non-cutaneous quality of life indexes, such as Dermatology Life Quality Index (DLQI), Skindex-26, and SF-36 [9–10, 13–17]. It should be noted that vitiligo is usually asymptomatic, so its effect on quality of life is much more related to psychological problems, such as lack of self-confidence [10, 12], unpleasant body images [19], unsuccessful social relationships [9], and lower quality of marital relations [13, 20], than the exclusive physical issues. It has been shown that patients with vitiligo are at risk of higher social discrimination and stigma [10, 14, 17, 19, 21].

A disease specific instrument for estimating quality of life in Vitiligo has been proposed by Lilly et al. at 2013; named as Vitiligo Quality of Life (VitiQoL) which emphasizes on three principal factors including: stigma, participation limitation, and behavior [4].

In Iran, there are some studies conducted on vitiligo quality of life, by the means of general indexes [18, 20, 22–25], all confirm poorer QOL especially in women [20] and poorer QOL compared with some other countries [22]. Assessment of quality of life of Iranian patients with vitiligo with a specific index has not been performed. The purpose of this study was to assess quality of life among Iranian vitiligo patients with VitiQoL.

## Methods

This study was conducted on 173 patients with vitiligo, in Razi Hospital, Tehran University of Medical Sciences (TUMS), Tehran, Iran, from April 2013 to September 2014. VitiQoL was used as the specific index for assessment of quality of life in vitiligo patients. The questionnaire consisted of 15 questions with a seven-point Likert scale (0-6). The final scores could range from 0 to 90, in which, patients with higher scores showed poorer quality of life.

Two bilingual physicians translated English version of VitiQoL questionnaire to Persian, then Persian version was translated to English by another bilingual person who had not read the original questionnaire (back to back translation). Afterward, the final version was compared with the original English version. Finally, a dermatologist, a community and preventive medicine specialist, and a psychiatrist, confirmed the Persian questionnaire.

VASI score was used to measure severity of the disease (Additional file 1) [6]. The scores of specific areas such as hand, foot, breast, and genitalia, also exposed and non-exposed areas, were calculated. “Disease progression” and “response to treatment” were assessed in questionnaire, based on the subjective opinion of patients regarding disease course in the previous month. The following two questions were asked respectively: “Did you experience any change in the severity and number of lesions within the previous month?” (Disease progression; improving, no change, slowly progressive, rapidly progressive); and, “How did your lesions respond to treatment in the previous month?” (Response to treatment; poor, moderate, appropriate). Previous psychological or other dermatological diseases were also asked from the patients.

For the pilot study, 25 patients who were referred to the phototherapy clinic of Razi Hospital and consented to participate in the study, were selected. These patients filled the VitiQoL questionnaire in the first session and 2–3 weeks later. In these two sessions, a dermatologist (KH) as the main investigator of the study, answered any questions or comments of the patients. She interviewed the patients regarding their demographic data and clinical characteristics, and examined disease extent and depigmentation degree based on VASI score.

After the pilot study, essential modifications were made based on the opinion of research team on findings (e.g. change the structure of complicated sentences to be more comprehensible or use the words that were more familiar and making sense of the questions). The research team concluded that the patients could not easily discriminate among 7 items in Likert; therefore, the Persian version was finalized as 5-item Likert scale questions (final scores ranged from 0 to 60) (Additional file 2). The main structure of the original questionnaire was not changed and no word was omitted or added. The final questionnaire was used for the second phase (the main study).

In order to estimate the mean score of VitiQoL based on the report by Lilly et al. [4], a sample of 139 patients was necessary. A single estimation of mean was used for sample size calculation. As the VitiQoL score could range from 0 to 90, considering a normal distribution, which included 6 standard deviations, a standard deviation of 15 was used for calculation of sample size (*n* = 97, if d = 3). To compensate for probable missing, we added 15 % to this calculated number; and finally 173 patients were enrolled in the study.

All patients who were at least 16 years old, did not have any other stigmatizing disorders or disabilities such as leprosy or facial deformities and were not mentally retarded or psychotic, also consented to participate in the study, were enrolled. Ninety patients were recruited from the general dermatology clinic and 83 patients from the phototherapy clinic, by convenience sampling. It should be mentioned that the patients recruited from phototherapy clinic were under treatment with narrow band UVB (NBUVB) and most of the patients of general clinic were on topical steroids or calcineurine inhibitors.

Data was entered in SPSS Statistics 22.0 and analyzed. Mean, median and standard deviation were used to describe quantitative data and Kolmogorov-Smirnov Z was used to assess normality of continuous variables. In the case of non-significant *P* values (*p* > =0.05), parametric *t*-test and analysis of variance (ANOVA) were used for normally distributed variables. For non-normal variables, Kruskal-Wallis H and Mann-Whitney *U* test were used based on the number of categories of the nominal variables. Pearson correlation coefficient was used for comparison of two normally distributed variables, and otherwise Spearman correlation was performed. McNemar test was used to assess any statistically significant changes in responses to dichotomous questions. For assessment of correlation between quality of life and VASI, Spearman correlation coefficient was used.

In 10 questionnaires, one or more questions were left blank, but none of them had more than 3 missing items. In analysis, we did not exclude any uncompleted filled questionnaire and the limited missing data, were replaced with 3 (mid-score).

In psychometric evaluation of VitiQoL questionnaire, confirmatory factor analysis was performed based on principal component analysis and Direct Oblimin (as the rotation method), eigenvalue > 1 and suppression of absolute value < 0.4.

Patients enrolled based on their consent and data were analyzed with respect to their confidentiality. The ethical committee of Tehran University of Medical Sciences approved the study. Authors had no conflict of interests to disclose.

## Results

In the pilot study, 13 women and 12 men were enrolled. The mean age was 31.96 years (SD = 12.21) with the range of 16–63 years. All of them were recruited from the phototherapy clinic. Analysis of pilot data showed a favorable face and content validity. In re-test, the reliability was confirmed by a high Cronbach alfa (0.956), which showed high internal consistency. The two series of Persian questionnaires were compared with rho-Spearman test and correlations were satisfactory. The Spearman correlation coefficient between the scores in the first time and in the second was 0.847. Concurrent validity of the Persian version of VitiQoL was confirmed by significant correlations between self-reported severity (question 16 of VitiQol) and VitiQoL scores (*r* = 0.463 and *p* < 0.05). Including all patients, Cronbach’s alpha of VitiQol was 0.914.

Demographic data and clinical characteristics of patients of the main study are shown in Table 1. Table 2 shows VASI and its relationship with clinical data of patients. Significant relationship between VASI and treatment group, duration of disease, education, type of disease (generalized > localized > segmental) and having another visible skin disorder in addition to vitiligo were found (P < 0.05). Skin phototype, gender, age, and concurrent or previous psychiatric problems did not show any significant relationship with VASI (p>0.05).

**Table 1:** Descriptive demographic data of patients with vitiligo

		General clinic (*n* = 90)		Phototherapy clinic (*n* = 83)		Total (*n* = 173)	
		n	%	n	%	n	%
Gender	Female	36	40	48	57.8	84	48.6
	Male	54	60	35	42.2	89	51.4
Age	16–19	13	14.9	15	18.1	28	16.2
	20–29	21	23.0	28	33.7	49	28.3
	30–39	34	37.9	22	26.5	56	32.4
	> = 40	22	24.1	18	21.7	40	23.1
Disease duration (years)	<5	46	52.9	17	20.5	63	36.4
	5- < 10	13	14.9	20	24.1	33	19.1
	10- < 15	13	13.8	24	28.9	37	21.4
	15- < 20	6	6.9	7	8.4	13	7.5
	> = 20	12	11.5	15	18.1	27	15.6
Education	Illiterate	4	4.4	0	0	4	2.3
	Grade 1–11	25	27.8	19	22.9	44	25.4
	Grade 12	38	42.2	38	45.8	76	43.9
	University education	23	25.6	26	31.3	49	28.3
Skin phototype (Fitzpatrick scale)	1,2	3	3.3	1	1.2	4	2.3
	3,4	74	82.3	79	95.2	153	88.4
	5,6	13	14.4	3	3.6	16	9.2
Type of disease	Generalized	76	84.4	72	86.7	148	85.5
	Localized	7	7.8	8	9.6	15	8.7
	Segmental	7	7.8	3	3.6	10	5.8
Disease progression	Improving	25	27.8	57	68.7	82	47.4
	No change	16	17.8	17	20.5	33	19.1
	Slowly progressive	33	36.7	7	8.4	40	23.1
	Rapidly progressive	16	17.8	2	2.4	18	10.4
Response to treatment	Poor	32	35.5	18	21.7	50	28.9
	Moderate	25	27.7	28	33.7	53	30.6
	Appropriate	28	31.1	37	44.6	65	37.6
	No treatment^a^	5	5.6	0	0	5	2.9
Previous or current psychiatric disease	Yes	7	7.8	10	12	17	9.8
	No	83	92.2	73	88	156	90.2
Another visible skin disorder other than vitiligo	Yes	3	3.3	6	7.2	9	5.2
	No	87	96.7	77	92.8	164	94.8

^a^Patients who were not on any treatment

**Table 2:** Vitiligo Area Scoring Index (VASI) and its correlation with demographic characteristic of patients with vitiligo

		N	Mean of VASI	Standard deviation of VASI	Median of VASI	*P* value
Total		173	8.62	13.09	3.8	
Treatment group	General Clinic	93	6.08	8.44	1.87	0.04
	Phototherapy Clinic	80	8.70	9.09	6.02	
Gender	Female	84	9.50	14.63	4.44	0.2
	Male	89	7.80	11.43	3.18	
Age	16–19	28	6.96	9.36	1.71	0.1
	20–29	49	7.71	11.86	4.38	
	30–39	56	7.56	11.85	3.13	
	> = 40	40	12.38	17.47	6.7	
Disease duration (years)	<5	63	3.61	5.56	1.54	<0.001
	5- < 10	33	7.96	8.97	3.75	
	10- < 15	37	11.10	14.78	4.56	
	15- < 20	13	15.34	10.70	12.87	
	> = 20	27	14.46	21.87	6.55	
Education	Illiterate	4	19.39	11.77	17.62	0.009
	Grade 1–11	44	10.54	16.01	6.33	
	Grade 12	76	9.12	13.68	3.22	
	University education	49	5.24	7.63	2.80	
Skin phototype (Fitzpatrick scale)	1,2	4	3.97	11.77	1.64	0.2
	3,4	153	8.46	13.32	3.75	
	5,6	16	11.32	12.17	4.94	
Type of disease	Generalized	148	9.92	13.73	5.54	<0.001
	Localized	15	0.77	1.35	0.30	
	Segmental	10	0.95	1.28	0.40	
Disease progression	Improving	82	7.49	8.51	4.90	0.6
	No change	33	11.11	21.40	3.42	
	Slowly progressive	40	10.13	13.82	3.87	
	Rapidly progressive	18	5.81	7.55	1.84	
Response to treatment	Poor	50	6.69	7.41	3.31	0.4
	Moderate	53	8.06	10.88	5.58	
	Appropriate	65	10.92	17.59	4.19	
	No treatment^a^	5	3.84	6.58	0.33	
Previous or current psychiatric disease	Yes	17	7.33	8.99	3.42	0.99
	No	156	8.76	13.48	4.25	
Another visible skin disorder other than vitiligo	Yes	9	2.52	2.76	1.59	0.04
	No	164	8.95	13.48	4.25	

^a^Patients who were not on any treatment

VASI (total and area’s specific) and its correlation with VitiQoL have been demonstrated in Table 3. It should be noted that in analytic evaluation of VASI and quality of life, data of three patients with nearly universal vitiligo from general dermatology clinic were excluded as outliers; since these patients had the most severe disease based on VASI, but their universal depigmentation caused the least prominent disease presentation, comparing to other patients with severe non-homogenous pigmentary lesions. Finally, the VASI data of 170 patients were analyzed.

The detail of QOL assessment is shown in Table 4. The mean VitiQoL score was 30.5 (range: 0–60, SD = 14.5, median = 31). Using principal component analysis and Direct Oblimin method, three factors from VitiQoL were extracted: participation limitation (question number 2-11, 14), stigma (question number 1, 15), and behavior (question number 8, 12, 13). Overall, women had poorer QOL (higher VitiQoL scores) than men. However, in subscale analysis of these three factors of VitiQoL, the difference was significant only for behavior factor (and not for participation limitation and stigma) (*p* = 0.02). The QOL (based on behavior factor) was better in patients recruited from the general clinic than the patients of phototherapy clinic (*p* = 0.001).

**Table 3:** Vitiligo Area Scoring Index (VASI) of total skin and area specific, and correlation of VASI and VitiQol

	Mean of VASI	Standard deviation of VASI	Median of VASI	Correlation between VASI and VitiQol
Total (*n* = 173)	8.62	13.09	3.8	
Excluding outliers (*n* = 170)	7.36	8.84	3.67	*p* = 0.015 *r* = 0.187
(Female = 82, Male = 88)				
Head and neck	0.52	1.12	1	*p* = 0.40
Upper limb	2.17	3.41	0.5	
Hand	0.66	0.73	0.3	
Lower limb	3.68	4.67	1	
Foot	1.6	1.22	0.5	
Trunk	2.86	5.48	0.5	
Non-exposed area	6.57	8.24	2.83	
Exposed-area	0.8	0.85	0.79	*p* = 0.75
Female	0.61	0.66	0.4	
Male	0.97	0.69	0.67	
Breast	1.44	0.73	0	
Female	0.55	0.4	0.9	*p* = 0.07 *r* = 0.417
Male	0.14	0.51	0	*p* = 0.8
Genitalia	0.14	0.26	0.3	*p* = 0.54
Female	0.2	0.1	0	
Male	0.2	0.13	0.6

**Table 4:** VitiQol and descriptive demographic characteristics of patients with vitiligo

		Number	Mean of VitiQol	Standard deviation of VitiQol	Median of VitiQol	*P* value
Treatment group	General Clinic	93	28.80	14.64	28.5	0.107
	Phototherapy Clinic	80	32.40	14.30	33	
Gender	Female	84	31.70	15.20	32.5	0.006
	Male	89	29.73	13.85	30	
Age	16–19	28	30.82	16.40	27.5	0.026
	20–29	49	34.32	12.43	36	
	30–39	56	29.23	14.25	27.5	
	> = 40	40	28.65	15.47	28.5	
Disease duration (years)	<5	63	27.60	14.95	24	0.037
	5- < 10	33	34.63	15.57	36	
	10- < 15	37	31.78	13.51	35	
	15- < 20	13	28.76	12.65	29	
	> = 20	27	32.74	13.39	32	
Education	Illiterate	4	37.00	13.44	38.5	0.667
	Grade 1–11	44	32.34	16.05	35	
	Grade 12	76	30.21	14.23	30	
	University education	49	29.81	13.68	28	
Skin phototype (Fitzpatrick scale)	1,2	4	34.50	17.71	29.5	0.408
	3,4	153	31.15	14.16	32	
	5,6	16	26.43	17.05	22	
Type of disease	Generalized	148	31.61	14.39	32	0.161
	Localized	15	28.20	13.87	27	
	Segmental	10	24.46	15.01	20	
Disease progression	Improving	82	31.82	14.38	32	0.296
	No change	33	32.42	14.17	34	
	Slowly progressive	40	26.95	15.68	25	
	Rapidly progressive	18	31.66	11.98	33	
Response to treatment	Poor	50	31.16	15.29	30	0.195
	Moderate	53	31.83	13.13	33	
	Appropriate	65	30.72	14.95	30	
	No treatment^a^	5	17.20	10.03	14	
Previous or current psychiatric disease	Yes	17	36.41	12.84	38	0.066
	No	156	29.91	14.58	30	
Another visible skin disorder other than vitiligo	Yes	9	22.77	11.02	20	0.102
	No	164	30.98	14.62	32	

^a^Patients who were not on any treatment

Patients who had disease duration of 5 years or less, and patients with 15–20 years disease duration, had better quality of life, although it was not statistically significant. Quality of life was better in more educated patients (*p* > 0.05). Patients without previous history of other visible cutaneous disorders and patients with lighter skin phototype had poorer QOL; but it wasn’t significant (*p* > 0.05).

Psychiatric problems were associated with poorer QOL, although the association was not significant (*p* = 0.06, *r* = 0.14). QOL was poorer in patients with more extensive disease, (generalized < localized < segmental), and the best QOL was observed in slowly progressive disorder, but none of them were statistically significant (*p* > 0.05). The QOLs of two treatment groups were not significantly different (*p* > 0.05).

There was a significant relationship between VASI score and quality of life, in a way that higher VASI scores were associated with poorer quality of life (*p* = 0.015 *r* = 0.187). Regarding VitiQoL subscales, the correlation with VASI was statistically significant for behavior factor (*p* = 0.01, *r* = 0.254). There were not any significant associations between VASI of specific areas (head and neck, genitalia or exposed areas) and QOL (*p* > 0.05). There were not any associations between VASI of breast and quality of life, although in women it was near to be meaningful (*p* = 0.07, *r* = 0.417); nevertheless, in the behavior factor subscale analysis, significant correlation was demonstrated (*p* = 0.03, *r* = 0.22).

## Discussion

With regard to relatively high prevalence of vitiligo in the world [1, 4], involvement of young people [2, 3] and high disease burden [9–16], evaluation of disease quality of life with a specific scale in countries with various cultural values could be worthwhile. VitiQoL is originally a questionnaire in English, which recently has been suggested for estimation of quality of life in vitiligo patients. In the present study, a Persian version of this scoring system was designed and evaluated.

Catucci et al. translated VitiQoL to Brazilian Portuguese (VitiQoL-PB) and performed cultural adaptation and validation [26]. in the original VitiQoL, one of the limitations of the study was no assessment of test-retest reliability. In Catucci study, 16 subjects (21 % of the sample) completed the VitiQoL-PB for the second time, confirming the high test-retest reliability with an intra-class correlation co-efficient (ICC) of 0.95 (95 % CI, 0.86 to 0.98) [26]. In our study 25 patients in pilot step were completed Persian version twice by 14–21 days interval. The reliability of the Persian version was confirmed by Cronbach alfa (0.956), which was similar to original VitiQoL Cronbach’s alpha (0.935) [4] and Brazilian Portuguese study (Cronbach alpha = 0.944) [26]. Correlations between self-reported severity and VitiQoL scores (convergent validity) in Persian version (*p* < 0.05, *r* = 0.463) was near to correlation coefficient of the primary study [4] (*p* < 0.05, *r* = 0.51) and Brazilian Portuguese one [26].

In this study, factor analysis revealed three distinct factors (participation limitation, stigma, and behavior) within vitiligo quality of life and that these factors were internally consistent. Also the original factor structure proposed by Lilly et al. was retained. The correlation between disease severity and QOL was comparable to the results of other studies [24]. Better life quality of patients in general clinic in our study is reasonable, because of less severity (usually <15–20 % body surface area (BSA)), and thus, lower VASI scores.

Poorer QOL in female patients based on behavior factor was logically predictable, since all over the world, aesthetic issues are more considerable in woman. They express a higher level of emotional burden, and the disease has strong impact on their self-steam. This result was compatible with Dolatshahi et al. study [20] to some extent, which demonstrated relationship between gender and QOL, so that more attention to this group is needed.

This study showed that younger patients had poorer quality of life, and 20–29 year-old patients experienced the worst life quality. It makes sense that cosmetic issues are more troublesome for the younger people; as they might have unmet needs of being equally considered, respected and welcome in occupational, social or emotional relationships. Disease duration less than 5 years and 15–20 years showed better quality of life, which might reflect higher hopes for successful treatment in early course of the disease or getting used to the disease in the long-term.

It was expected that patients with university educations might be more aware of the nature of problem and less worried about any potential consequences, hence have better quality of life. Nevertheless, this study did not show a significant association between educational level and QOL, perhaps due to the preponderance of other educational categories in comparison with university education.

According to the literature, vitiligo is considered as both cause and result of some psychiatric problems such as anxiety and depression [8–17]; thus history of other psychiatric problems could potentially add to the burden of this disorder. As some vitiligo patients with psychiatric problems might not be aware of their own disorder and the disorder could have been undiagnosed, the association may be even stronger. This study did not find any associations between skin phototype and quality of life, possibly due to the small number of patients with phototype 1 and 2 in Fitzpatrick scale classification.

There were not any association between VASI of exposed areas (face, hand, foot) and quality of life. Results were compatible with the Linthorst [15] and Dolatshahi [20] studies. However it was in contrast to the study by Ongenae et al. [27], which showed quality of life improves with camouflage. It should be noted that only 15 % of patients had vitiligo involvement limited to non-exposed areas. The study showed that patients with higher educational level and those suffering from another skin disorder other than vitiligo, have lower VASI score, which could be interpreted as alertness and precocious referral of more educated persons and higher sensitivity to skin appearance among patients with other concurrent cutaneous disorders. This was to be expected that the mean VASI of patients in phototherapy clinic be higher than general clinic, since dermatologists usually suggest phototherapy for patients with involvement of more than 15–20 % of BSA. Also it was reasonable to find higher VASI scores in patients with more prolonged disorder, due to probable progressive nature of the disease.

### Limitations

Sampling was performed in a referral university hospital, which may not be representative of all spectrums of vitiligo patients in the society. Nevertheless, this center (Razi Hospital) is the largest dermatology center of Iran and this might decrease the chance of sampling bias. Further multi-centric studies are needed to validate the results.

Minor psychological problems did not prohibit patients’ enrolment as that exclusion might have led to the selection of a sub-sample who were potentially healthier than normal Iranian population [28]. Moreover, based on previous studies, these psychological problems are rather higher in patients with vitiligo [8].

Moreover, non-significance of some associations might have been due to lack of enough power considering multiple comparisons. In this study, adjustment for *P* values was not considered and p less than 0.05 was considered significant.

In Persian version, the original 7-item Likert score of VitiQoL was reduced to 5, due to the difficulty of the respondents to differentiate between very close measures of frequency (such as sometimes and occasionally).

### Recommendations

Future multi-centric and community-based studies would be useful to improve the validity and reliability of Persian version of VitiQoL. Online filling of the questionnaire can be applied in future research. Finally, designing a trial to assess and improve patients’ information regarding the disease characteristics and a before-after evaluation of QOL would be a novel idea for future studies. It would be an honor for the authors of this paper to provide other researchers with Persian version of VitiQoL for further studies on vitiligo patients’ quality of life and the questionnaire is available upon their written request.

## Conclusion

In this study, the Persian version of a specific index for estimating life quality of patients with vitiligo was developed. Except in patients with nearly all body surface area involved, the quality of life was worse when the disease was more severe. Life of these patients is obviously affected by presence of depigmented skin lesions, especially in women, younger adults and those with disease duration of 5–10 years. Finally, quality of life should be considered as one of the important outcome measures of treatments in vitiligo patients.

## Additional files

Additional file 1: Vitiligo Area Scoring Index (VASI) for evaluation of disease extent and severity [6]. (JPG 213 kb) Additional file 2: Persian version of Vitiligo Quality of Life (VitiQoL) [4]. (PDF 535 kb)
