# Supplementary figures and images for: Quality of life in patients with vitiligo: a cross-sectional study based on Vitiligo Quality of Life index (VitiQoL)
Source: Health Qual Life Outcomes. 2016 Jun 7;14:86. doi: 10.1186/s12955-016-0490-y (PMC4897932; doi:10.1186/s12955-016-0490-y)

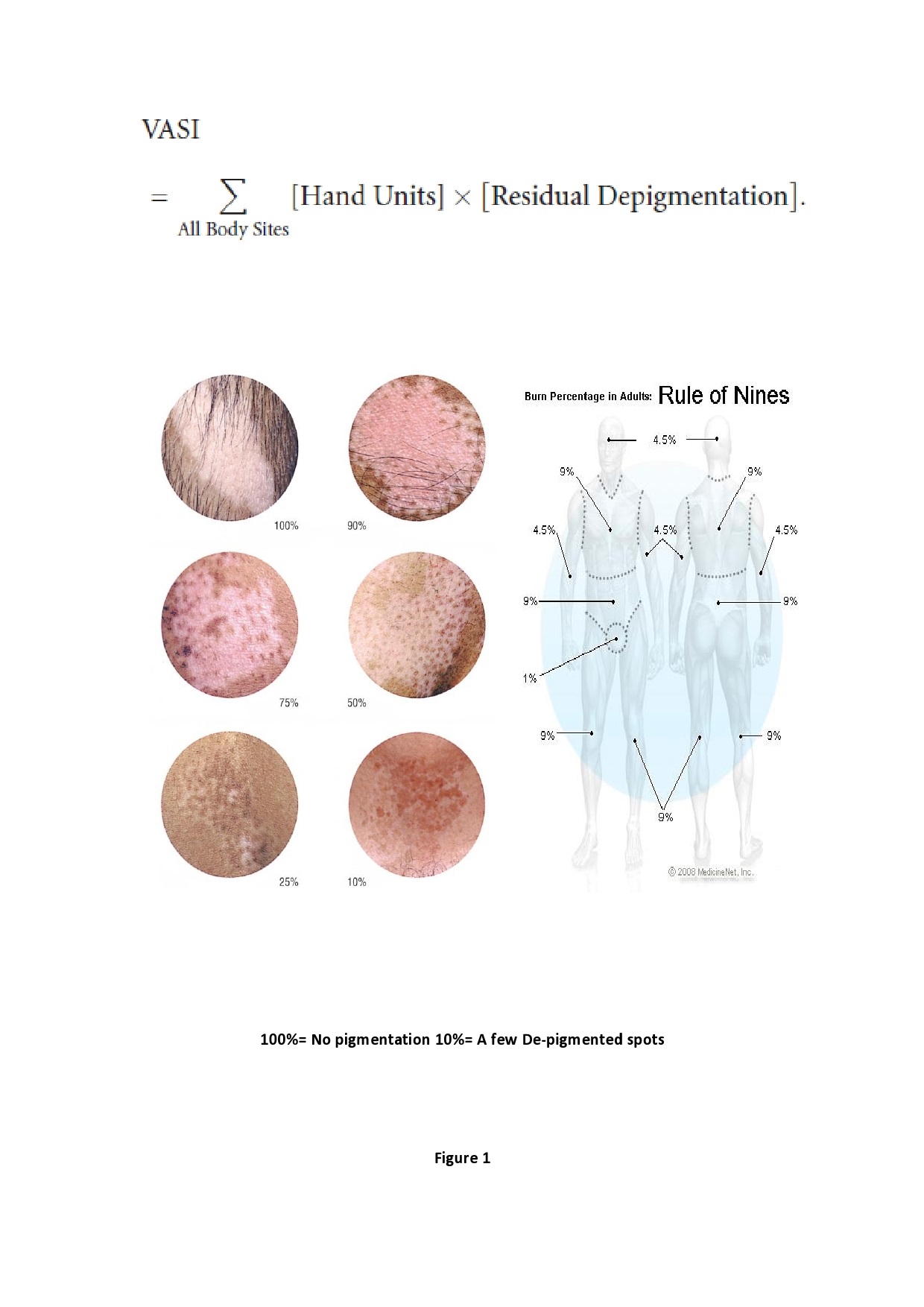

Supplement: Additional file 1: — Vitiligo Area Scoring Index (VASI) for evaluation of disease extent and severity [6]. (JPG 213 kb) [file 12955_2016_490_MOESM1_ESM.jpg]
