# Supplementary material for: Quality of life in patients with vitiligo: a cross-sectional study based on Vitiligo Quality of Life index (VitiQoL)
Source: Health Qual Life Outcomes. 2016 Jun 7;14:86. doi: 10.1186/s12955-016-0490-y (PMC4897932; doi:10.1186/s12955-016-0490-y)
Supplement: Additional file 2: — Persian version of Vitiligo Quality of Life (VitiQoL) [4]. (PDF 535 kb) [file 12955_2016_490_MOESM2_ESM.pdf]

پرسشنامه بررسی کیفیت زندگی در بیماران مبتلا به ویتیلیگو

هدف از این پرسشنامه بررسی این موضوع است که وضعیت پوست شما طی ماه گذشته تا چه حد بر زندگی شما تاثیر گذاشته است؟

در طی ماه گذشته:

	به هیچ وجه		گاهی		همیشه
**۱-آیا ظاهر پوستتان باعث ناراحتی شما شده است؟**	☐۰	☐۱	☐۲	☐۳	☐۴
**۲-آیا وضعیت پوستتان باعث سرخوردگی تان شده است؟**	☐۰	☐۱	☐۲	☐۳	☐۴
**۳-آیا وضعیت پوستتان ابراز احساسات درونی را برای شما مشکل کرده است؟**	☐۰	☐۱	☐۲	☐۳	☐۴
**۴-آیا وضعیت پوستتان بر فعالیت های روزانه شما تأثیر گذاشته است؟**	☐۰	☐۱	☐۲	☐۳	☐۴
**۵-آیا به هنگام صحبت با دیگران نگران آن بوده اید که آن ها ممکن است چه فکری درباره ی شما بکنند؟**	☐۰	☐۱	☐۲	☐۳	☐۴
**۶-آیا شما نگران آن بوده اید که دیگران در شما ایرادی پیدا کنند؟**	☐۰	☐۱	☐۲	☐۳	☐۴
**۷-آیا شما به خاطر وضعیت پوستتان خجالت زده یا شرمنده شده اید؟**	☐۰	☐۱	☐۲	☐۳	☐۴
**۸-آیا وضعیت پوستتان بر لباس هایی که می پوشید تأثیر گذاشته است؟**	☐۰	☐۱	☐۲	☐۳	☐۴
**۹-آیا وضعیت پوستتان روی فعالیت های اجتماعی یا تفریحی تان تأثیری داشته است؟**	☐۰	☐۱	☐۲	☐۳	☐۴
**۱۰-آیا وضعیت پوستتان در سلامت شما از نظر احساسی تأثیر داشته است؟**	☐۰	☐۱	☐۲	☐۳	☐۴
**۱۱-آیا وضعیت پوستتان در سلامت کلی جسمی شما تأثیر گذاشته است؟**	☐۰	☐۱	☐۲	☐۳	☐۴
**۱۲-آیا وضعیت پوستتان در فعالیت های آرایشی- پیرایشی شما تأثیر گذاشته است (مثل مدل مو یا استفاده از لوازم آرایش)؟**	☐۰	☐۱	☐۲	☐۳	☐۴
**۱۳-آیا وضعیت پوستتان در روش محافظت شما در برابر آفتاب در ایام تفریح تأثیر گذاشته است (مانند محدود کردن مدت زمان ماندن در آفتاب در ساعات حداکثر نور، به دنبال سایه گشتن، استفاده از کلاه، لباس آستین بلند یا شلوار)؟**	☐۰	☐۱	☐۲	☐۳	☐۴
**۱۴-آیا وضعیت پوستتان بر شانس شما برای پیدا کردن دوستان جدید تأثیر گذاشته است؟**	☐۰	☐۱	☐۲	☐۳	☐۴
**۱۵-آیا نگران پیشرفت یا انتشار بیماری پوستی تان به سایر نقاط بدنتان بوده اید؟**	☐۰	☐۱	☐۲	☐۳	☐۴

	بسیار خفیف			متوسط			بسیار شدید
**۱۶-حس می کنید در حال حاضر بیماری تان چه قدر شدید است؟**	۰	۱	۲	۳	۴	۵	۶

۱۷.آیا تمام موارد فوق را پاسخ داده اید؟ بلی☐ خیر☐
